# Laparoscopic radical cystectomy with pelvic re-peritonealization: the technique and initial clinical outcomes

**DOI:** 10.1186/s12894-018-0424-6

**Published:** 2018-12-12

**Authors:** Qiang Cao, Pengchao Li, Xiao Yang, Jian Qian, Zengjun Wang, Qiang Lu, Min Gu

**Affiliations:** Department of Urology, the First Affiliated Hospital of Nanjing Medical University, 300 Guangzhou Road, Nanjing, 210029 China

**Keywords:** Laparoscopic radical cystectomy, Bowel function recovery, Re-peritonealization, Postoperative ileus

## Abstract

**Background:**

Delayed bowel function recovery and postoperative ileus are relatively serious complications of laparoscopic radical cystectomy (LRC). Our study aimed to determine whether performing pelvic re-peritonealization reduces the incidence of these complications.

**Methods:**

Clinical data of 78 patients who had undergone LRC with pelvic re-peritonealization from August 2015 to December 2017 were retrospectively collected and compared with those of 92 patients who had undergone LRC alone between January 2013 and July 2015 in our institution. Differences in duration of surgery, estimated blood loss, time to recovery of bowel function, the complications of intestinal and blood vessel injury, and incidence of postoperative ileus between the two groups were analyzed.

**Results:**

Baseline characteristics such as age, sex and BMI were balanced between the two groups. There were no significant differences in duration of surgery (*P* = 0.072), estimated blood loss (*P* = 0.717), or incidence of intestinal obstruction (*P* = 0.225) between the two groups. Interestingly, patients who had undergone pelvic re-peritonealization recovered bowel function more rapidly than those had not (2.79 d vs. 3.72 d, *P* = 0.001). Additionally, hospitalization stay was significantly shorter for patients with re-peritonealization than for those without (5.46 d vs. 6.68 d, *P* = 0.029).

**Conclusions:**

Compared with LRC alone, LRC with pelvic re-peritonealization as described in the present study had comparable perioperative complications, but was associated with more rapid gastrointestinal recovery and shorter hospitalization stay.

## Background

Bladder carcinoma (BC), which is characterized by high risks of recurrence and mortality, is among the fifth most common malignancies worldwide [1] . Open radical cystectomy (ORC) with pelvic lymphadenectomy and subsequent urinary diversion remains the standard treatment for muscle-invasive and high-grade non-invasive bladder carcinoma [2, 3]. However, ORC is considered one of the most invasive of urological surgeries and has significant perioperative complications [4, 5]. Before 1970, the perioperative complication rate was approximately 35% with a mortality rate of nearly 20% [6]. With medical advances, the mortality rate has decreased significantly, however, the postoperative morbidity of this surgery remains as high as 30% [7, 8]. Laparoscopic radical cystectomy (LRC), a minimally invasive treatment for bladder cancer, has achieved surgical outcomes that are comparable to those of ORC, but with fewer perioperative complications. Therefore, it has been widely substituted for ORC by urologists.

**Fig. 1:**
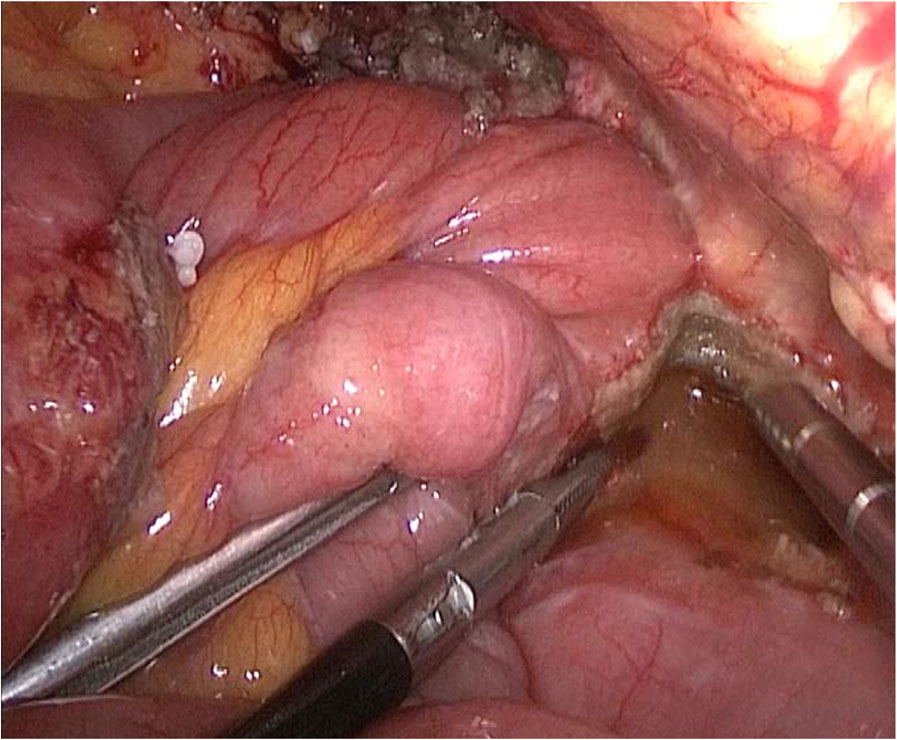
An illustrative case of a 65-year-old woman who developed ileus four days postoperatively. Laparoscopic examination revealed that the intestinal tract had adhered to the pelvic wound surface, creating angulation of the bowel

Perioperative complications related to the gastrointestinal tract are among the most common complications following radical cystectomy and urinary diversion [9]. Delayed recovery of bowel function and bowel obstruction may occur in the early postoperative period, the latter resulting from either paralytic ileus or mechanical obstruction [9]. Even in patients undergoing LRC, these complications are still problematic. Additionally, adhesive ileus may occur at an early stage after surgery as a result of the intestinal tract adhering to the wound in the pelvic cavity. Figure 1 shows a typical example in which the intestinal tract adhered to the pelvic wound surface leading to angulation of the bowel followed by mechanical obstruction four days postoperatively, which may have been prevented if the pelvic peritoneum had been preserved. Although possible risk factors such as age, BMI and various perioperative variables have been suggested, as have preventive techniques for early postoperative paralytic ileus [10], there are few reports of techniques for minimizing the incidence of gastrointestinal complications. Here, we introduce a technique of pelvic re-peritonealization following LRC with pelvic lymphadenectomy and subsequent urinary diversion aimed at enhancing recovery of bowel function and reducing the incidence of postoperative ileus, report our preliminary experience with this procedure in 78 patients, and retrospectively compare the outcomes with those of 92 patients who had undergone LRC alone.

## Methods

From August 2015 to December 2017, 78 consecutive patients with muscle invasive bladder cancer underwent LRC with pelvic re-peritonealization (LRC-PR) and standard lymph node dissection in our department. For comparison with these patients, another 92 patients who had undergone LRC between January 2013 and July 2015 in our center were identified and matched to the LRC-PR group by age, sex and BMI. All patients had undergone pelvic magnetic resonance/enhanced computed tomography, cystoscopy, and biopsy preoperatively. The clinical characteristics of these patients are presented in Table 1. The study was approved by the Institutional Review Board of the first affiliated hospital of Nanjing Medical University. Written informed consent was obtained from all the participants.

**Table 1:** The clinical characteristics and perioperative parameters of patients received LRC with or without pelvic re-peritonealization

Variables	LRC-PR	LRC	*P* ^*#*^
Patients, n	78	92	
Age	58.1 ± 8.35	57.5 ± 7.29	0.709
Gender			
Male	56 (71.8%)	68 (73.9%)	0.757
Female	22 (29.2%)	24 (26.1%)	
BMI (kg/m^2^)	23.05 ± 3.48	24.34 ± 3.13	0.149
Total operative time	263.5 ± 36.5	244.1 ± 41.2	0.072
Estimated blood loss	312.3 ± 120.1	301.8 ± 91.3	0.717
Major Complications ^*^	–	–	–
Bowel recovery time	2.79 ± 1.07	3.72 ± 0.93	0.001
Bowel obstruction	4 (5.1%)	9 (9.8%)	0.225
Hospitalization stay, d	5.46 ± 1.82	6.68 ± 2.21	0.029

^*^ No major complications such as intestinal and blood vessels injury was observed in both groups

^*#*^ Student t test for continuous variables; the chi-square test for categorical variables

*LRC-PR* laparoscopic radical cystectomy with pelvic re-peritonealization, *LRC* laparoscopic radical cystectomy

**Fig. 2:**
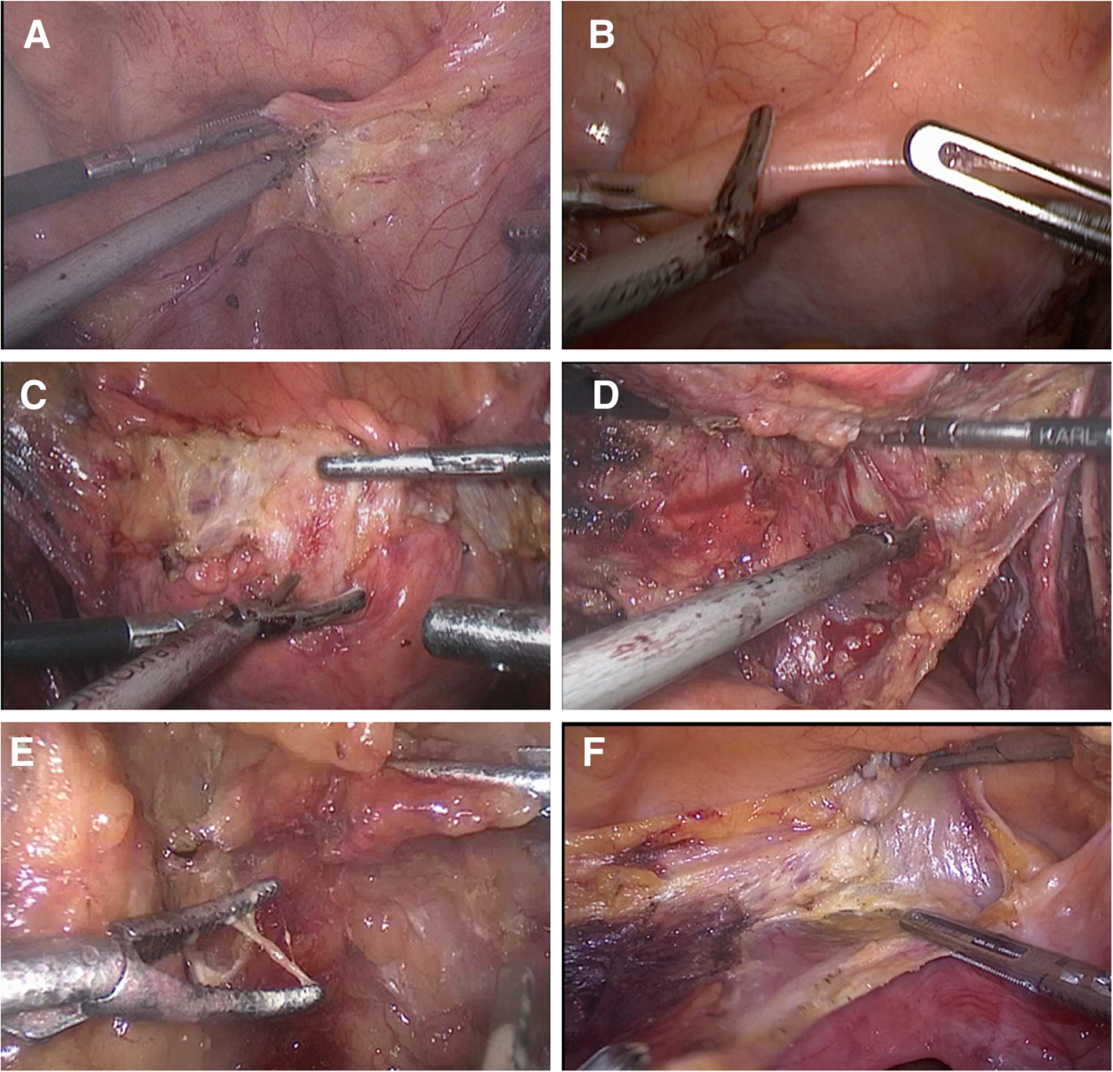
The procedure for dissection of the peritoneum: (**a**) The peritoneum is incised transversely at the dome of the bladder. (**b**) The peritoneum is incised longitudinally. (**c**–**e**) Dissection of the peritoneum from the dome of the bladder in a posterolateral direction. (**f**) Removal of the peritoneum covering the dome of bladder because of difficulty in separating it from the bladder

All the patients were administered general anesthesia and placed in the supine position with the head tilted down at an angle of 30°. Laparoscopic ports were introduced as previously described [11]. In the LRC alone group, only the peritoneum covering the iliac blood vessels was preserved. In the LRC-PR group, the procedure was conducted as follows. During the operation, the superficial peritoneum of the external iliac vessel was incised, dissected medially along the vas deferens to the seminal vesicle, and preserved. The contralateral vas deferens and seminal vesicle were dissected using the same procedure. The peritoneum was then incised transversely at the dome of bladder (Fig. 2a), after which it was separated from the dome in a posterolateral direction (Fig. 2c-e). The peritoneum covering the dome of bladder was removed with the bladder if it was difficult to separate it from the bladder (Fig. 2f). The bladder was then dissected dorsally from the dome. During the dissection, the seminal vesicle could be easily identified. Denonvilliers’ fascia was then incised, followed by dissection of the lateral ligaments of the bladder. A standard pelvic lymphadenectomy was performed after removal of the bladder. If the preserved peritoneum covering the anterior wall of the bladder blocked the surgical field during the procedure, a Hem-o-lok clip was used to temporarily fix it to the ipsilateral abdominal wall.

Another way of preserving the peritoneum was to incise the peritoneum covering the bladder longitudinally from the ventral to the dorsal side (Fig. 2b). The peritoneum at the dome of the bladder was not preserved if it was difficult to dissect (Fig. 2f). During the dissection, careful attention was paid to avoiding incising the muscle layer of bladder. When dissecting the left part, the surgeon lifted the peritoneum to the left, using an ultrasonic scalpel to isolate the peritoneum along the adipose tissue while the assistant pulled the bladder to the opposite side to maintain tension. The same principles were adopted as for dissection of the right side of peritoneum.

**Fig. 3:**
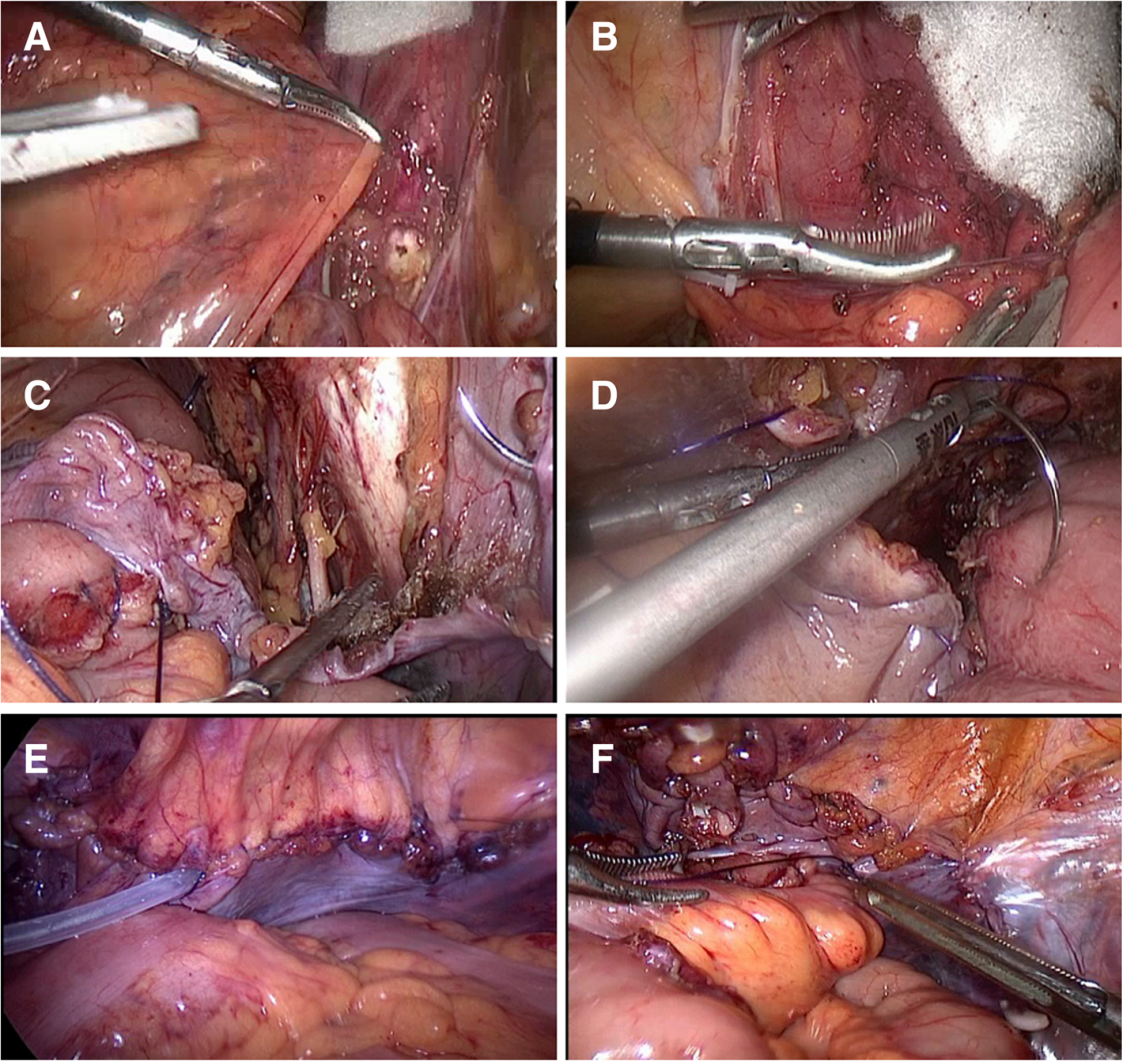
Closing of the preserved peritoneum. (**a**, **b**) Closing of the lateral peritoneum. (**c**) Barbed surgical thread is used to continuously suture the pelvic peritoneum. (**d**) In patients undergoing construction of an orthotopic ileal neobladder, the preserved peritoneum is sewn using interrupted sutures to the neobladder. (**e**, **f**). The end result of pelvic peritonealization during laparoscopic radical cystectomy with ureterocutaneostomy (**e**) and construction of an orthotopic ileal neobladder (**f**)

After performing cystectomy and lymphadenectomy, the reperitonealization was achieved as follows (Fig. 3). First, the lateral peritoneum was closed along the external iliac vessels using a Hem-o-lok or titanium clip from the cranial to the caudal end (Fig. 3a, b). The pelvic peritoneum was then continuously sutured using barbed surgical thread to seal the pelvic cavity (Fig. 3c). In patients who had undergone ureterocutaneostomy, a drainage tube was inserted into the pelvic cavity though a space between the peritoneal stitches (Fig. 3e). In patients who had had an orthotopic ileal neobladder constructed, the preserved peritoneum was sewn with interrupted sutures to the neobladder to seal the pelvic space (Fig. 3d-f) with the aim of reducing the risk of internal herniation.

All operations were performed by the same laparoscopic surgical team (QL, PL and QC). The following perioperative data were collected: duration of operation, time to bowel recovery, estimated blood loss, hospital stay, incidence of bowel and vessel injury, and incidence of bowel obstruction. Because few patients had ileal conduits or orthotopic ileal neobladders constructed, only patients who had undergone LRC with ureter–abdominal ostomies were included in the present study. Statistical analysis was performed using Student’s *t*-test for continuous variables and the χ^2^ test for categorical variables. All data are reported as means and ranges, with *P* values less than 0.05 considered to denote statistical significance.

## Results

All procedures were successfully performed laparoscopically without the need for open conversion. Relevant clinical characteristics of the two groups are presented in Table 1. There were no significant differences in the distribution of age, sex or BMI between the two groups (all *P* > 0.05). At 263.5 mins, the mean duration of surgery was slightly longer in the LRC–PR than the LRC group (244.1 mins); however this difference is not statistically significant (*P* = 0.072). The mean estimated blood loss was 312.3 mL and 301.8 mL in the LRC-PR and LRC groups, respectively; these values are comparable (*P* = 0.717). No major complications such as bowel or vessel injury occurred in either group. The time to bowel recovery was 2.79 days in the LRC-PR group, which is significantly shorter than that in the LRC group (3.72 days) (*P* = 0.001). The incidence of bowel obstruction was 5.1% (four patients) in the LRC–PR and 9.8% (nine patients) in the LRC group; this difference is not significant (*P* = 0.225). The four patients with bowel obstruction in the LRC–PR group recovered with conservative treatment, whereas six of the nine patients with bowel obstruction in the LRC group underwent laparoscopic surgery for adhesive ileus; an example is shown in Fig. 1. Additionally, hospital stay was significantly shorter in the group with re-peritonealization than in the group with LRC alone (5.46 d vs.6.68 d, *P* = 0.029).

## Discussion

We here report our experience of the first clinical use of a novel technique for laparoscopic radical cystectomy with pelvic re-peritonealization. We demonstrated that the patients who underwent this procedure had more rapid postoperative recovery of gastrointestinal function and shorter hospital stay than those who underwent LRC alone.

Intestinal complications are common after radical cystectomy. The most frequent of these complications is postoperative ileus, which is often responsible for extended hospital stays and delayed recovery after surgery. The pathophysiology of postoperative ileus is complex and not yet fully understood. The many factors that have been recognized to be associated with postoperative ileus can be categorized as preoperative, intra-operative, and postoperative [10]. Several factors that may be associated with postoperative recovery of gastrointestinal function have been addressed in enhanced recovery after surgery (ERAS) protocols, which include evidence-based steps to optimize postoperative recovery and shorten hospital stay, mainly through expediting recovery of gastrointestinal function.

The incidence of paralytic ileus is reportedly significantly reduced by implementing ERAS protocols in patients undergoing radical cystectomy [9, 12]. However, most items addressed in ERAS protocols are pre- or post-operative; there are few reports on improving surgical techniques to reduce the incidence of gastrointestinal complications. For instance, Rivas et al. [12] reported a study using an ERAS protocol in patients with bladder cancer undergoing laparoscopic radical cystectomy. Of the 17 items included in their protocol, only four were intraoperative, namely using short-acting anesthetic agents, epidural anesthesia/analgesia, normothermia, and maintenance of normovolemia. Although none of these items involve modification of surgical technique, they found the complication of paralytic ileus occurred significantly less frequently in patients undergoing the ERAS protocol. In a review of gastrointestinal complications in patients subjected to enhanced recovery protocols, Djaladat et al. found that general anesthesia, opiate use, surgical trauma and bowel manipulation, bowel preparation, postoperative nasogastric tube, perioperative goal-directed fluid therapy, and chewing gum to be associated with reduced time to recovery of gastrointestinal function and a lower rate of paralytic ileus [9]. However, none of these items are related to surgical technique.

In the present study, during the procedure, we performed pelvic peritonealization after the cystectomy and lymphadenectomy. Patients who underwent this modified technique had significantly earlier bowel recovery, a lower incidence of paralytic ileus, and shorter hospital stays than those who did not. It has been suggested that neutrophil infiltration of the muscularis mucosae and subsequent ileus are related to the presence of inflammatory mediators released in response to surgical stimulation [13]. Additionally, exposure of the bowel to urine or transudation during intra- or post-operatively also induce an inflammatory reaction in the muscularis. In patients who undergo pelvic re-peritonealization, the pelvic space is sealed by peritonealization, confining intra- and post-operative exudates such as urine, blood, interstitial fluid, and lymphatic fluid to the pelvic cavity and thus facilitating their drainage through a drainage tube, reducing irritation of the intestines and possibly expediting recovery of bowel function. As previously mentioned, adhesion of the intestinal tract to the pelvic wound surface can lead to angulation of the bowel and consequent mechanical obstruction; this occurs relatively frequently in clinical practice (Fig. 1). Theoretically, pelvic re-peritonealization may also reduce the risk of intestinal adhesion and late adhesive intestinal obstruction. Additionally, without pelvic re-peritonealization, the intestine may prolapse into the pelvic cavity, which can also result in intestinal angulation and consequently obstruction. However, there is a risk of cutting bladder muscle during the procedure for preservation of the peritoneum. Therefore, we consider this procedure contraindicated in patients with greater than T2 disease and in slender patients with insufficient extraperitoneal adipose tissue.

The initial procedure for constructing an ileal conduit included closure of all mesenteric and peritoneal defects to minimize potential internal herniation; however, recent advances in laparoscopic and robotic techniques have often resulted in these defects being left open [14] [15]. Such defects can lead to small bowel entrapment and consequently obstruction, which carries a significant morbidity and mortality. The incidence of internal herniation is reportedly not affected by prior abdominal radiation or peritonitis; however, sewing the cut peritoneal edge to the conduit to reperitonealize it [14, 15] and closure of any mesenteric defects may prevent internal herniation [16]. In the present study, we used interrupted sutures to sew the preserved peritoneum to the conduit in patients undergoing construction of an orthotopic ileal neobladder; these patients’ bowel function recovered rapidly and none of them developed postoperative paralytic ileus. However, the incidence of late intestinal complications requires further investigation.

Several limitations of the present study need to be addressed. First, this was a small study, especially regarding patients who underwent construction of an orthotopic ileal neobladder. Second, this was a single-center trial and would thus inevitably have been subject to selection bias. Third, the duration of follow-up was relatively short, allowing investigation of perioperative complications only. Late complications such as adhesive intestinal obstruction occur frequently in patients who have undergone LRC; long-term follow-up is therefore needed.

## Conclusions

We here introduce a new technique for pelvic re-peritonealization during LRC. We found that this procedure is feasible and safe. Although, the procedure was slightly time consuming, it could effectively promote the bowel function recovery and may facilitate the ERAS program for patients with bladder cancer. Therefore, we suggest that LRC with pelvic re-peritonealization should be considered in suitable patients if surgically available available. However, larger studies and a multicenter prospective randomized controlled trial are required to confirm our results.

## Abbreviations

BC: Bladder carcinoma
BMI: Body Mass Index
EBL: Estimated blood loss
ERAS: Enhanced recovery after surgery
LRC: Laparoscopic radical cystectomy
LRC-RP: LRC with pelvic re-peritonealization
ORC: Open radical cystectomy
